# Incidental Adult Optic Pathway Glioma and Cerebral Venous Aneurysm in Neurofibromatosis Type 1 With Prior Synovial Sarcoma: A Multidisciplinary Challenge

**DOI:** 10.7759/cureus.111367

**Published:** 2026-06-23

**Authors:** Rania Chakir, Boutaina Agdi, Sara Harbaj, Amina Majdi, Karima Nouni, Lachgar Amine, Hanane Elkacemi, Tayeb Kebdani, Khalid Hassouni

**Affiliations:** 1 Radiation Therapy, National Institute of Oncology, Rabat, MAR; 2 Radiotherapy, National Institute of Oncology, Faculty of Medicine and Pharmacy, University Mohamed V, Rabat, MAR; 3 Radiotherapy, National Institute of Oncology, Rabat, MAR; 4 Radiation Oncology, National Institute of Oncology, Rabat, MAR; 5 Radiation Oncology, National Institute of Oncology, Faculty of Medicine, Mohammed V University, Rabat, MAR

**Keywords:** ganglion cell layer (gcl), neurofibromatosis type 1, optical coherence tomography (oct), optic pathway glioma, ras/mapk, synovial sarcoma, vasculopathy, venous aneurysm

## Abstract

Neurofibromatosis type 1 (NF1) is a multisystemic disorder predisposing to various neoplasms and vasculopathies. While optic pathway gliomas (OPGs) are hallmarks of pediatric NF1, their incidental discovery in adults, especially when associated with persistent unidentified bright objects (UBOs), mesenchymal malignancies, and rare venous anomalies, is exceptional.

A 49-year-old female with NF1 and a history of cervical synovial sarcoma underwent routine oncological surveillance. Brain MRI incidentally revealed a WHO Grade 1 OPG, multiple persistent UBOs in the brainstem and cerebellar peduncles, and a focal parietal venous aneurysm. Despite being asymptomatic, multimodal ophthalmological imaging was performed. Optical coherence tomography (OCT) showed significant asymmetric axonal loss. The left eye (OS) exhibited marked peripapillary retinal nerve fiber layer (pRNFL) thinning (69 µm, p < 0.01). Notably, while the right eye (OD) had normal pRNFL thickness (83 µm), macular ganglion cell layer (GCL) analysis detected early focal neuro-retinal suffering (54 µm minimum thickness). Automated perimetry confirmed functional correlation in both eyes.

This case highlights the "multi-tumor challenge" in NF1, suggesting a high-penetrance phenotype driven by RAS/MAPK hyperactivation. The persistence of UBOs into the fifth decade and the discovery of a rare venous aneurysm likely due to medial dysplasia underscore a unique systemic vasculopathy. Unlike sporadic adult OPGs, NF1-associated forms often follow an indolent course, justifying a "watch-and-wait" strategy.

Managing complex adult NF1 phenotypes requires a multidisciplinary approach. This case demonstrates that GCL-OCT can serve as a sensitive "early warning system" for axonal loss before clinical symptoms arise, facilitating non-invasive surveillance in genetically vulnerable individuals.

## Introduction

Neurofibromatosis type 1 (NF1), also known as Von Recklinghausen’s disease, is an autosomal, dominant, multisystemic disorder caused by a germline mutation in the NF1 gene on chromosome 17q11.2. This gene encodes neurofibromin, a key negative regulator of the RAS/MAPK signaling pathway, an intracellular signaling cascade that strictly regulates cell growth, division, and differentiation. Its loss of function leads to constitutive cellular proliferation, predisposing patients to a wide spectrum of both benign and malignant neoplasms [1]. While NF1 is characterized by high penetrance, its clinical expressivity remains remarkably variable, even within the same family.

Optic pathway glioma (OPG) is one of the defining features of NF1, occurring in approximately 15-20% of the pediatric population [2,3]. In children, NF1-associated OPGs follow a classic clinical course: they are typically low-grade pilocytic astrocytomas presenting before the age of seven that often exhibit benign behavior, remaining stable or even undergoing spontaneous regression after adolescence. In stark contrast, de novo adult OPGs in the general population are sporadic, highly symptomatic, aggressive, and high-grade malignancies. The incidental discovery of a quiescent, completely asymptomatic OPG in adulthood within an NF1 patient represents an atypical presentation that mimics the latency of benign pediatric forms rather than the aggressive nature of sporadic adult high-grade gliomas. Managing these adult lesions raises significant diagnostic and therapeutic challenges, particularly regarding the differentiation between indolent NF1-associated lesions and aggressive sporadic high-grade gliomas [4-6].

The diagnostic monitoring of these lesions has been advanced by optical coherence tomography (OCT), which allows for the detection of subclinical structural changes and axonal loss before global nerve fiber layer thinning or definitive functional visual field deficits manifest [7,8]. Beyond neuro-oncological manifestations, NF1 is associated with a distinct risk of mesenchymal malignancies, such as synovial sarcoma, and a specific vasculopathy [9]. Although arterial involvements, such as stenoses, occlusions, or aneurysms, are well-documented [10,11], the involvement of the venous system remains exceptionally rare in the literature. This venous wall fragility is thought to result from medial dysplasia, defined as the structural weakening of the vascular wall's middle layer (tunica media) due to neurofibromin deficiency in vascular smooth muscle cells [12]. Furthermore, patients can present with unidentified bright objects (UBOs), which are focal T2-hyperintense white matter abnormalities corresponding pathologically to areas of intramyelinic vacuolar degeneration [13-16].

While existing literature details these manifestations in isolation, this case addresses a unique clinical knowledge gap by reporting the exceptional, simultaneous coexistence of a high-grade mesenchymal malignancy (prior cervical synovial sarcoma), an incidental adult OPG, a rare subcutaneous venous aneurysm, and the atypical persistence of multiple UBOs well into the fifth decade of life. This specific multisystemic and multitumor phenotype is distinct from previously reported adult NF1 cohorts, which are almost universally symptomatic or dominated by classic malignant peripheral nerve sheath tumors (MPNSTs). Ultimately, this case report highlights a rare phenotypic spectrum of the disease driven by high penetrance, emphasizing the critical necessity of lifelong multidisciplinary monitoring and the pivotal role of advanced multimodal imaging in managing adult NF1 manifestations [17,18].

## Case presentation

A 49-year-old female patient, followed for NF1 based on established diagnostic criteria, including café-au-lait macules, cutaneous neurofibromas, and axillary freckling, presented with a significant history of cervical synovial sarcoma diagnosed in 2008. The sarcoma was managed with surgical resection followed by adjuvant radiotherapy (total dose of 30 Gy, delivered in 15 fractions of 2 Gy using an anterior-posterior opposed field technique and two oblique fields).

Magnetic resonance imaging (MRI)

While maintaining stable, long-term oncological remission of the sarcoma, the patient underwent a routine brain MRI as part of the systematic neurological surveillance protocol recommended for NF1 patients (Figure 1). This imaging incidentally revealed a low-grade left OPG involving the intracanalicular and pre-chiasmatic segments, measuring 7.06 mm in maximum diameter with a longitudinal extension of 12.9 mm. This lesion was closely associated with a dilated and tortuous venous structure in continuity with a branch of the superficial temporal vein (Figure 2). These radiological findings are highly suggestive of a subcutaneous venous aneurysm. The presence of this vascular malformation, coexisting with multiple cutaneous neurofibromas and the OPG, underscores the complex, multisystemic nature of NF1 in this patient.

**Figure 1:**
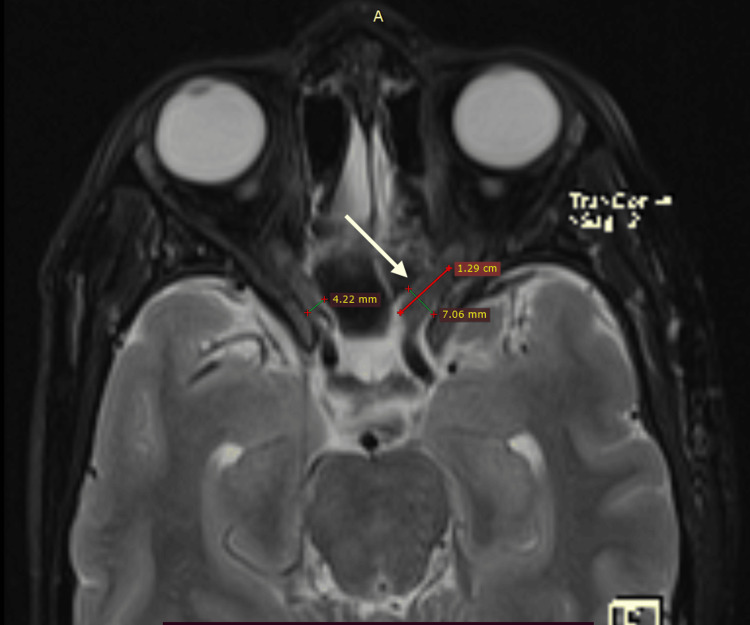
Axial T2-weighted brain MRI showing a left-sided optic pathway glioma (OPG) There is a significant fusiform enlargement of the left optic nerve involving the canalicular and pre-chiasmatic segments, measuring 7.06 mm in diameter and 1.29 cm in longitudinal extension (highlighted by the white arrow). For comparison, the patient's normal right optic nerve measures 4.22 mm. These findings are highly characteristic of a low-grade glioma associated with neurofibromatosis type 1 (NF1).

**Figure 2:**
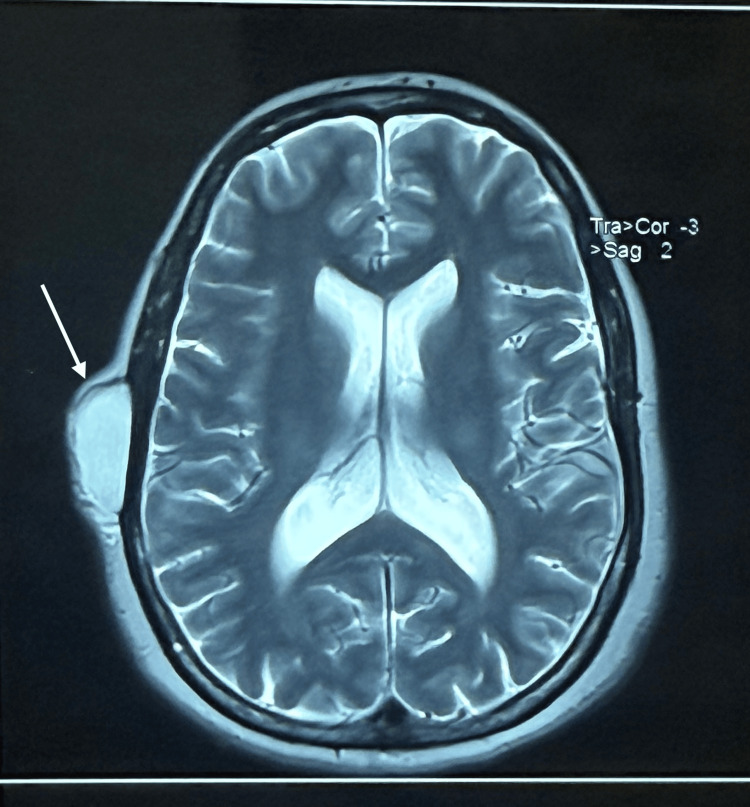
Axial T2-weighted brain MRI showing a subcutaneous venous aneurysm Magnetic resonance imaging (MRI) of the patient reveals a multi-loculated subcutaneous formation in the right parietal region, characterized by high T2 signal intensity (indicated by the white arrow ). The lesion measures 41 x 16 x 46 mm and demonstrates direct continuity with a dilated superficial venous structure, a finding highly suggestive of a subcutaneous venous aneurysm. Furthermore, the wider intracranial sequences identify punctate hyperintensities within the white matter. These are consistent with T2-hyperintense foci, commonly referred to as unidentified bright objects (UBOs), which are characteristic neuroimaging hallmarks of the patient’s underlying neurofibromatosis type 1 (NF1).

Ophthalmological evaluation

At presentation, the neuro-ophthalmological examination was entirely normal, confirming the subclinical and asymptomatic nature of the lesion. The best-corrected visual acuity (BCVA) was 10/10 (20/20) in both eyes, and color vision was normal with no detected dyschromatopsia. Pupillary light reflexes were normal and symmetrical, with no relative afferent pupillary defect (RAPD). Intraocular pressure (IOP) was within normal limits in both eyes, measuring 14 mmHg in the right eye and 15 mmHg in the left eye.

As shown in Figure 3, color fundus photography was normal bilaterally, revealing sharp optic disc margins with a normal cup-to-disc ratio and no evidence of papilledema or optic disc atrophy.

**Figure 3:**
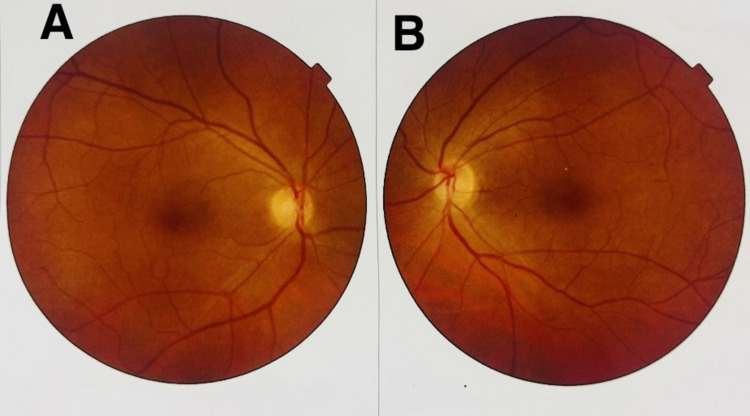
Color fundus photography of the right eye (A) and left eye (B) of our patient A: The photograph shows clear ocular media with well-defined optic disc margins and a normal cup-to-disc ratio. Retinal vasculature and macula appear normal with no visible pathological changes. B: The photograph shows a preserved optic disc appearance and regular retinal distribution. No edema, exudates, or pigmentary changes are visualized.

However, given the patient's history of NF1 and previous high-grade malignancy, a comprehensive structural evaluation using OCT and automated perimetry was performed to rule out subclinical optic pathway involvement. A detailed summary of these baseline clinical parameters, functional tests, and quantitative multimodal imaging data is systematically compiled in Table 1.

**Table 1:** Comprehensive baseline ophthalmological parameters and quantitative multimodal imaging data BCVA: best-corrected visual acuity; IOP: intraocular pressure; RAPD: relative afferent pupillary defect; PRNFL: peripapillary retinal nerve fiber layer; GCL-IPL: ganglion cell layer-inner plexiform layer; DB: decibels; µm: micrometers Note the isolated, statistically significant thinning of the minimum GCL-IPL complex in the right eye (54 µm) in contrast to a strictly normal average pRNFL profile (83 µm) and preserved functional assessments, demonstrating early subclinical optic pathway involvement.

Clinical & Imaging Parameters	Right Eye (OD)	Left Eye (OS)	Clinical Significance / Normative Reference
Best-Corrected Visual Acuity (BCVA)	10/10 (20/20)	10/10 (20/20)	Intact central vision bilaterally
Intraocular Pressure (IOP)	14 mmHg	15 mmHg	Normal range (10–21 mmHg)
Color Vision (Ishihara Plate Test)	15/15	15/15	No dyschromatopsia detected
Relative Afferent Pupillary Defect (RAPD)	Absent	Absent	No conductive optic nerve pathway lesion
Optic Disc Fundus Examination	Normal appearance	Normal appearance	Absence of papilledema or macroscopic atrophy
Automated Visual Field (Mean Deviation)	-1.12 dB (Normal)	-0.98 dB (Normal)	No functional visual field loss detected
Average pRNFL Thickness	83 µm (Green/Normal)	91 µm (Green/Normal)	Preserved global axonal profile
Minimum GCL-IPL Complex Thickness	54 µm (Red/Thinning)	78 µm (Green/Normal)	Subclinical focal structural loss (Right Eye)
Clinical Follow-up Duration	12 months	12 months	Documented functional & radiological stability

Structural and functional correlation

The functional evaluation of the patient’s left eye through automated perimetry (30-2 threshold test) revealed significant impairment, which directly correlated with the structural damage observed on OCT. The grayscale map and pattern deviation plots highlighted a meaningful reduction in retinal sensitivity, characterized by a dense superior arcuate scotoma (Figure 4). These findings were quantified by a mean deviation (MD) of -5.50 dB and a pattern standard deviation (PSD) of 2.60 dB. Despite these deficits, the test demonstrated high reliability, showing minimal fixation losses (2/14) and false-positive/negative rates well below acceptable clinical thresholds.

**Figure 4:**
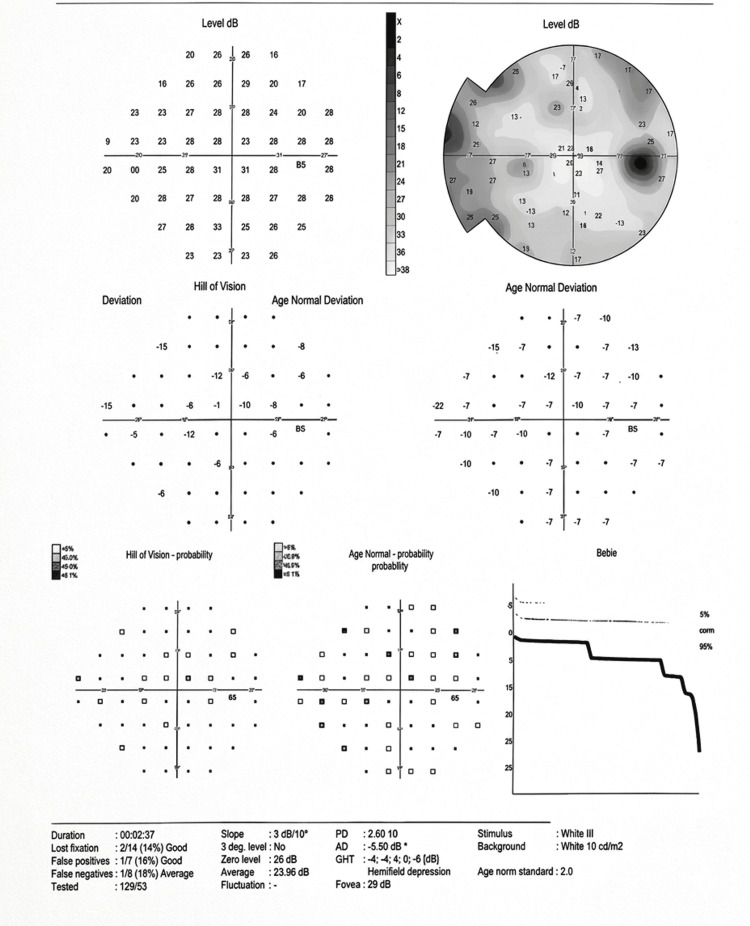
Automated static perimetry (30-2) of the left eye (OS) The grayscale map and deviation plots demonstrate a dense superior arcuate scotoma with an average deviation (AD) of -5.50 dB and a pattern deviation (PD) of 2.60 dB. The Glaucoma Hemifield Test (GHT) indicates a hemifield depression. Reliability indices are noted (fixation losses: 2/14, false positives: 14%, false negatives: 18%).

A detailed analysis of the cumulative defect curve (Bebie curve) provided further insight into the nature of this visual loss. The initial segment of the curve remained stable and closely aligned with the upper normative percentiles, demonstrating remarkable preservation of diffuse retinal sensitivity. However, the curve subsequently exhibited a pronounced downward stepwise shift, culminating in a sharp drop. This characteristic biphasic profile provided definitive diagnostic evidence of a deep, localized focal deficit rather than a generalized depression of the visual field, correlating with the mechanical compression of specific axonal bundles caused by the left OPG.

These functional results correlated with structural data obtained from the pRNFL analysis (Figure 5), revealing a significant structural asymmetry of 49% between both eyes. In the left eye, pathological thinning was pronounced, with an average pRNFL thickness of 69 µm (red zone), specifically affecting the superior and inferior quadrants. Conversely, the right eye exhibited a structurally preserved average pRNFL thickness of 83 µm (green zone) and a remarkably preserved functional visual field, with an MD of -3.31 dB and a PSD of 1.43 dB (Figure 6). Relying solely on these standard peripapillary pRNFL measurements and standard perimetry would have led to the conclusion of a perfectly normal right eye, missing early axonal involvement.

**Figure 5:**
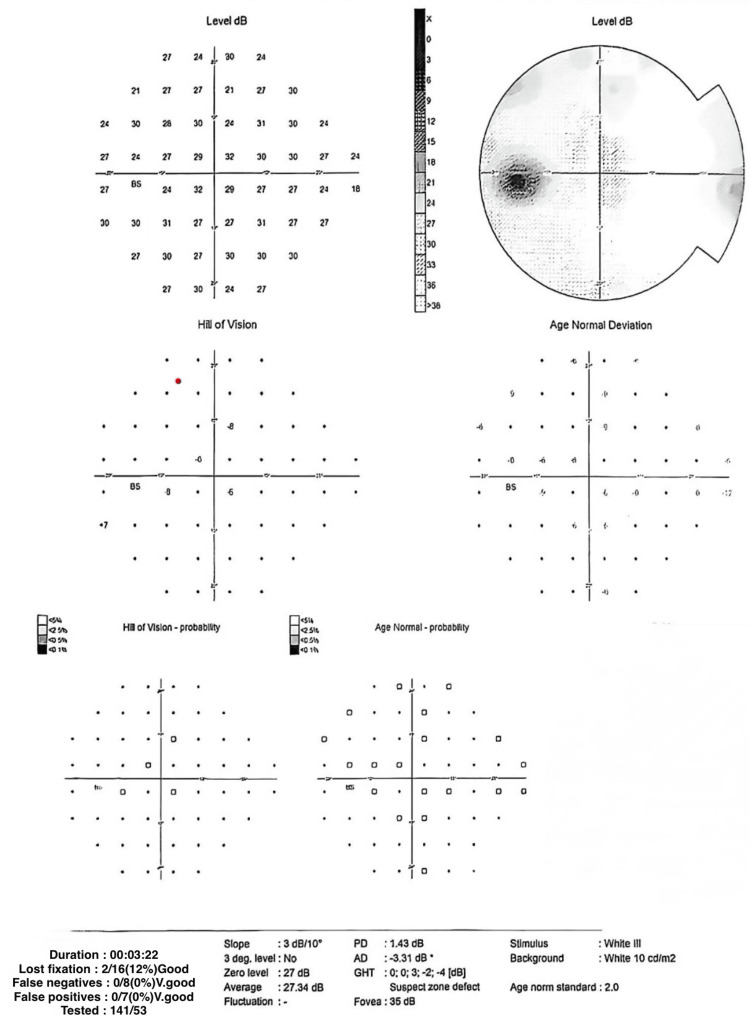
Automated visual field (VF) test: right eye (OD) The test demonstrates a preserved functional visual field with an average deviation (AD) of -3.31 dB and a pattern deviation (PD) of 1.43 dB. Only minor, non-specific depressions are visible, confirming the absence of chiasmatic extension. Reliability indices are excellent (fixation losses: 2/16, false positives: 0%, false negatives: 0%).

**Figure 6:**
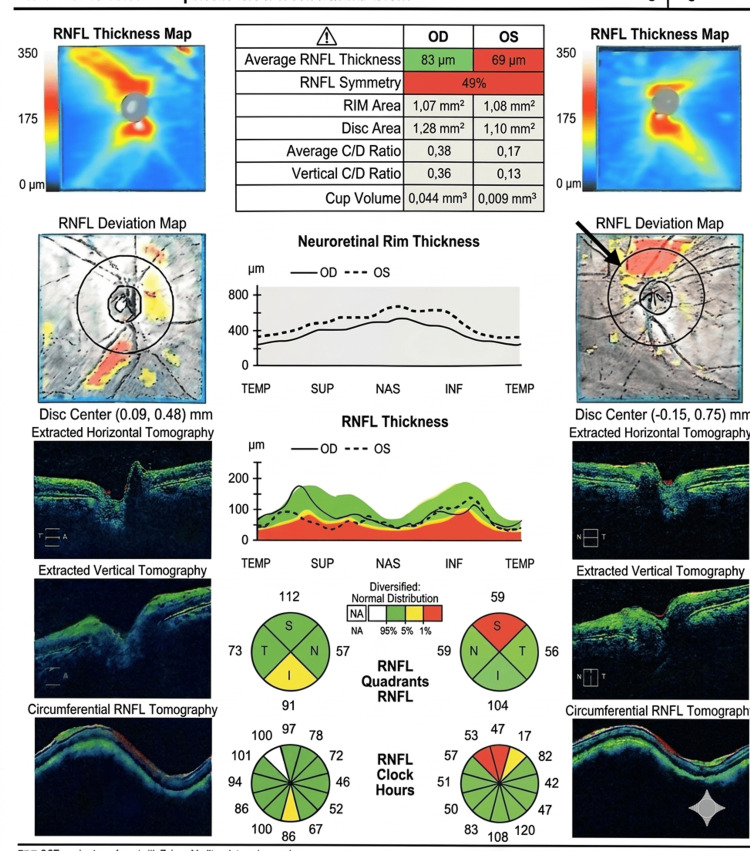
Optical OCT of the pRNFL The analysis reveals significant structural asymmetry between both eyes. The left eye (OS) demonstrates severe thinning of the pRNFL with an average thickness of 69 µm, while the right eye (OD) shows a preserved average thickness of 83 µm within the normal normative range. The RNFL symmetry index is markedly reduced at 49%. Tomograms provide a detailed cross-sectional view of the optic disc, showing a stable cup-to-disc ratio despite axonal loss in the left eye. The black arrow highlights the focal area of significant axonal loss on the RNFL deviation map of the left eye. OD: right eye (oculus dexter); OS: left eye (oculus sinister); pRNFL: peripapillary retinal nerve fiber layer; OCT: optical coherence tomography

Early detection via macular GCL analysis

To overcome the diagnostic limitations of standard peripapillary measurements, a macular ganglion cell layer (GCL) analysis was performed (Figure 7). Despite a normal peripapillary retinal nerve fiber layer (pRNFL) thickness and a well-preserved visual field in the right eye, GCL thickness mapping and sectorial analysis highlighted a focal zone of significant thinning, reaching a minimum thickness of 54 µm (red zone). This early, localized decrease in GCL thickness is thought to represent a potential early structural marker of retrograde transsynaptic axonal degeneration, suggesting subclinical neuronal loss prior to the onset of global nerve fiber layer thinning or functional visual field deficits.

**Figure 7:**
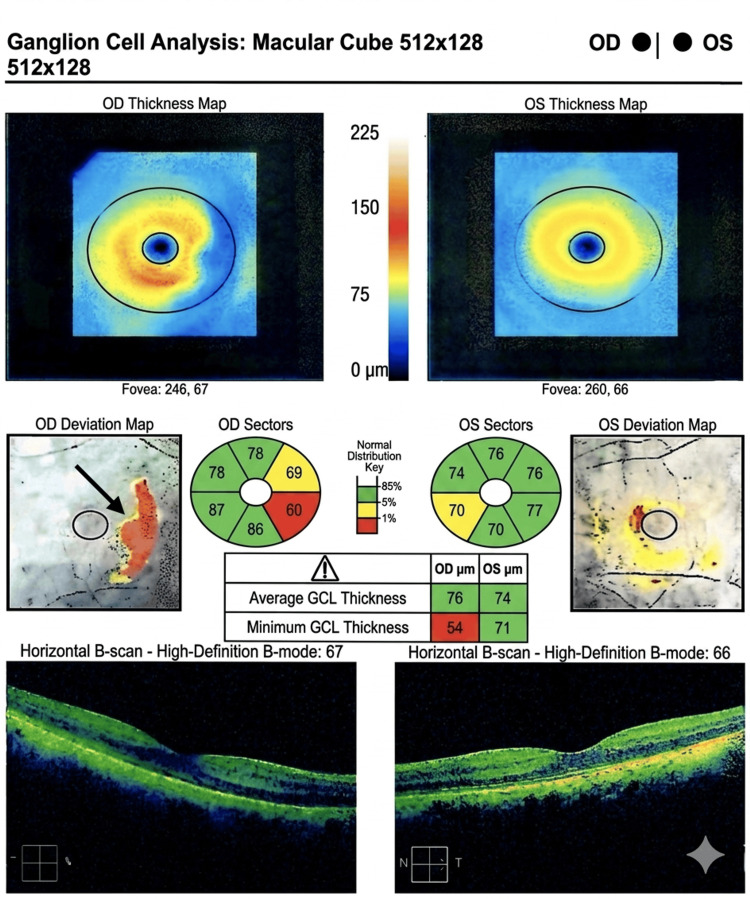
Macular GCL analysis OCT macular cube scan focusing on the ganglion cell-inner plexiform layer (GCIPL). In the right eye (OD), despite a normal average pRNFL thickness, the thickness map and sector analysis reveal a focal area of significant thinning, with a minimum GCL thickness of 54µm. The black arrow specifically highlights this prominent area of focal neuronal loss on the OD deviation map. The left eye (OS) demonstrates a less pronounced alteration in the minimum thickness parameter, 71 µm, though sector-specific thinning remains present. Deviation maps highlight the topographical distribution of inner retinal neuronal loss. OD: right eye (oculus dexter), OS: left eye (oculus sinister), GCL: ganglion cell layer, pRNFL: peripapillary retinal nerve fiber layer, OCT: optical coherence tomography

Finally, the analysis of the total macular thickness, from the internal limiting membrane to the retinal pigment epithelium (ILM-RPE) (Figure 8), confirmed well-preserved foveal contours in both eyes, with central subfield thicknesses of 275 µm in the right eye and 261 µm in the left eye. The structural integrity of the outer retinal layers explains the preservation of the patient's visual acuity despite the presence of the tumor. This precise anatomical compartmentalization emphasizes that pathological lesions in optic pathway gliomas associated with NF1 may be selectively limited to the inner retinal layers (GCL and RNFL), making early macular GCL screening a valuable tool for systemic surveillance.

**Figure 8:**
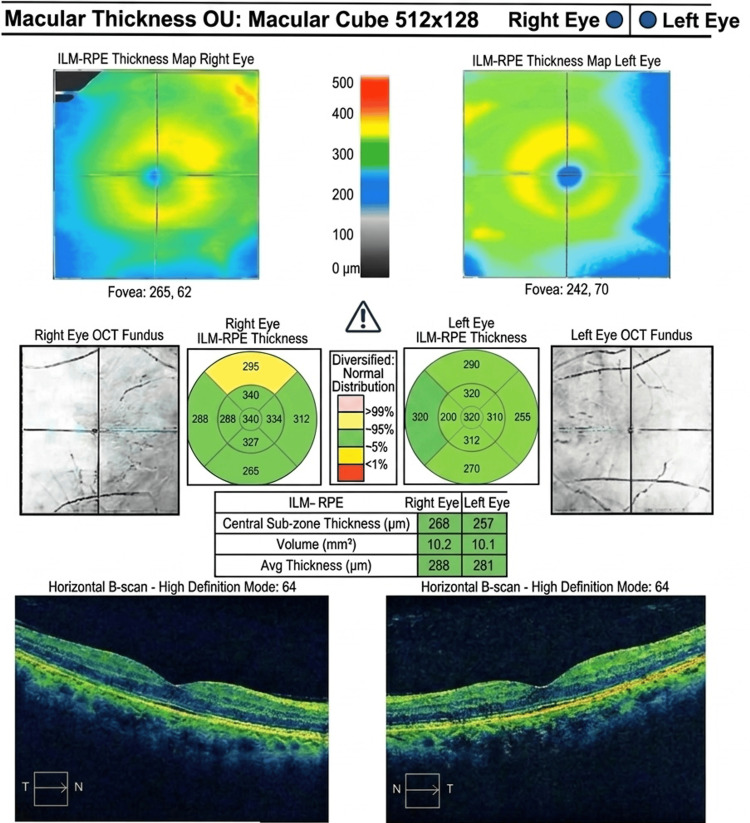
Macular thickness analysis OCT macular cube scan (512x128) measuring total retinal thickness from the internal limiting membrane (ILM) to the retinal pigment epithelium (RPE). Both thickness maps demonstrate a well-preserved foveal contour, with central subfield thicknesses of 275 µm in the right eye (OD) and 261 µm in the left eye (OS). Most sectors remain within the normative database range, indicating that the outer retinal layers are structurally intact. OD: right eye (oculus dexter), OS: left eye (oculus sinister), ILM: internal limiting membrane, RPE: retinal pigment epithelium, GCL: ganglion cell layer, RNFL: retinal nerve fiber layer, OCT: optical coherence tomography

Patient timeline and follow-up outcomes

The patient's clinical journey followed a structured, multistep sequence. Following the diagnosis and successful management of a cervical synovial sarcoma in 2008, she remained in complete oncological remission. A low-grade left OPG and a parietal subcutaneous venous aneurysm were incidentally discovered during routine neurological surveillance.

To confirm radiological stability, sequential neurological follow-up was performed, confirming the structural stability of the left OPG, with no growth or mass effect over a 12-month surveillance period. The right parietal subcutaneous vascular anomaly was confirmed using targeted magnetic resonance venography (MRV) sequences, which clearly demonstrated direct continuity with the superficial temporal venous branch, without intracranial extension or deep venous involvement.

Table 2 provides a timeline of the patient's history and presentation.

**Table 2:** Structured timeline of the patient’s clinical history and diagnostic sequence Chronological overview mapping the patient's long-term oncological evolution following treatment for cervical synovial sarcoma, the subsequent incidental multi-systemic neuro-ophthalmic and vascular discoveries during routine surveillance, and the established radiological stability of the optic pathway glioma.

Date / Period	Clinical Event & Diagnostic Sequence
2008	Diagnosis of cervical synovial sarcoma; treated with surgery and adjuvant radiotherapy (30 Gy).
2008-2026	Long-term surveillance confirming complete, stable oncological remission of the sarcoma.
May 2026	Routine brain MRI revealing incidental left OPG, persistent UBOs, and right parietal venous aneurysm.
May 2026	Multimodal ophthalmic workup (OCT, GCL analysis, Automated Perimetry) showing subclinical axonal loss.
Follow-up	MRV confirmation of the venous aneurysm and documentation of 12-month OPG radiological stability.

## Discussion

The diagnostic dilemma of adult OPG

OPGs are primarily a pediatric pathology, accounting for 10-15% of supratentorial tumors in children, and are closely linked to NF1 [1,2]. In adults, the literature classically distinguishes two entities: sporadic forms, which are often aggressive and high-grade, and NF1-associated forms, which tend to be more indolent [3]. Our case diverges from the usual clinical presentation due to its incidental discovery and completely asymptomatic nature in a 49-year-old patient. Indeed, in the largest cohort of adult OPGs analyzed to date by O'Connor et al. [4], nearly all patients (99.3%) presented with visual impairment at the time of diagnosis. Our case thus illustrates an atypical, indolent presentation of the pathology in adults, mimicking the quiescent behavior more commonly observed in pediatric NF1 cohorts.

Structural imaging via OCT and GCL analysis

Traditionally, pRNFL thinning has been used as the standard marker for monitoring axonal loss. The literature has demonstrated a robust correlation between pRNFL thinning and functional decline, validating OCT as an indispensable monitoring tool [5]. However, our results highlight the complementary sensitivity of the macular GCL complex.

In our patient's right eye, while the pRNFL appeared within the normal normative range (83 µm) (Figure 6), GCL analysis identified a focal area of thinning with a minimum thickness of 54 µm (Figure 7). This finding aligns with clinical data suggesting that macular GCL thinning can serve as a sensitive indicator for detecting subclinical optic pathway involvement, sometimes preceding measurable pRNFL alterations [6]. In this clinical context, the localized drop in GCL thickness has been proposed in current literature as a potential early structural marker of retrograde transsynaptic axonal degeneration. This framework suggests a mechanism of subclinical neuronal loss that may be identified before manifesting as global nerve fiber layer thinning or definitive functional visual field deficits, thereby offering a valuable window for non-invasive surveillance.

Multitumor phenotype and genomic instability

The association between NF1 and soft tissue sarcoma is exceptional. Kalyany et al. reported that these tumors often occur earlier in NF1 patients and exhibit increased aggressiveness [7]. Unlike classic nerve sheath tumors (MPNST), the emergence of a synovial sarcoma reflects an expanded mesenchymal tumor spectrum. At the molecular level, the literature indicates that the loss of function in the NF1 gene leads to the constitutive activation of the RAS/MAPK signaling pathway [8]. This pathway hyperactivation is thought to promote genomic instability, a vulnerability that facilitates the emergence of tumors from different cell lineages (glial and mesenchymal). The clinical presentation observed here, combining a history of high-grade sarcoma and an incidental low-grade glioma, is consistent with descriptions of a high-penetrance phenotype. This atypical association underscores the importance of ongoing surveillance in a genetically vulnerable background, particularly when considering the potential long-term tissue impact of prior adjuvant radiotherapy.

Venous vasculopathy: an underrecognized manifestation

The discovery of an isolated cerebral venous aneurysm adds a rare dimension to this report. While arterial abnormalities, such as stenoses, occlusions, or Moyamoya syndrome, are well-documented in NF1 [9], the involvement of the venous system remains underrepresented in the literature. This venous wall fragility may result from medial dysplasia related to neurofibromin deficiency in vascular smooth muscle cells [10]. Despite a perceived low risk of spontaneous rupture, the lack of specific data in the NF1 population justifies, according to the principles of Lasjaunias et al., regular non-invasive surveillance via magnetic resonance angiography (MRA) or MRV [15].

Persistence of UBOs in adulthood

The presence of UBOs is a major neuroradiological hallmark of NF1, detected in approximately 70% to 80% of the pediatric population [11]. Pathologically, these lesions correspond to areas of intramyelinic vacuolar degeneration and typically show spontaneous regression after adolescence, becoming increasingly rare beyond the second decade of life [12]. In the present case, the persistence of multiple UBOs in a 49-year-old patient (Figure 2) represents an atypical neuroradiological presentation. While the literature generally describes their disappearance in adulthood, some longitudinal studies suggest that certain lesions, particularly those located in the thalamus or the corpus callosum, may persist significantly longer than those in other regions [13]. The recognition of these foci is crucial in older patients to avoid a misdiagnosis of ischemic vascular disease or inflammatory demyelinating disorders, such as multiple sclerosis [12,13]. This is especially important as UBOs characteristically lack mass effect, do not show perilesional edema, and do not demonstrate contrast enhancement [12,13].

Management and multidisciplinary surveillance strategies

The management of NF1 necessitates a proactive, multidisciplinary approach involving specialists in oncology, neurology, ophthalmology, and radiation oncology. Specific surveillance recommendations include the following.

Ophthalmologic monitoring: Regular examinations are necessary to monitor OPG structural status, with stable, asymptomatic adult cases managed via conservative surveillance protocols [1,4].

Vascular and systemic vigilance: Regular clinical assessments and blood pressure monitoring are critical for detecting NF1-related vasculopathy or associated lesions [9,11].

Targeted imaging: MRI/MRV remains the standard for the follow-up of suspected intracranial tumors, stable lesions, and underlying vascular anomalies [1,10].

To further illustrate the distinctiveness of our clinical observation, we have conducted a comparative analysis between the classical data reported in the literature and the specific features identified in our patient. This comparison highlights how our case diverges from the typical pediatric presentation of NF1, particularly regarding the late-onset incidental discovery of the optic pathway glioma, the persistence of radiological markers into late adulthood, and the atypical nature of the associated vascular and mesenchymal pathologies. The main differences are summarized in Table 3.

**Table 3:** Comparison between classical literature and the findings in our case OPG: optic pathway glioma; OCT: optical coherence tomography; pRNFL: peripapillary retinal nerve fiber layer; GCL: ganglion cell layer; UBOs: unidentified bright objects; MPNST: malignant peripheral nerve sheath tumor

Feature	Classical Literature Findings	Findings in the Present Case	References
Optic Pathway Glioma (OPG) Age	Primarily a pediatric pathology, diagnosed before the age of 7 in 75% of cases.	Incidental discovery in an adult patient.	[1,2,4]
OPG Clinical Behavior	Often symptomatic, presenting with vision loss, proptosis, or endocrine dysfunction.	Completely asymptomatic with stable clinical and neuro-ophthalmological status.	[2,4]
Imaging Sensitivity (OCT)	Peripapillary retinal nerve fiber layer (pRNFL) thinning is the traditional gold standard for monitoring axonal loss.	Macular ganglion cell layer (GCL) thinning demonstrated higher sensitivity, appearing before pRNFL alterations.	[5,6]
UBOs Evolution	Unidentified Bright Objects (UBOs) typically regress spontaneously or disappear after the age of 20.	Persistent and multiple UBOs well into adulthood.	[11-13]
Vasculopathy Type	Predominantly arterial involvement, including stenoses, occlusions, Moyamoya syndrome, or arterial aneurysms.	Isolated subcutaneous venous aneurysm (right parietal region) driven by medial dysplasia.	[9,10]
Tumor Spectrum	Strong classical association with malignant peripheral nerve sheath tumors (MPNST) or optic gliomas.	Atypical coexistence with a prior history of high-grade cervical synovial sarcoma.	[7,8]

## Conclusions

In conclusion, this case illustrates the complex, multisystemic nature of NF1 in an adult patient. The incidental discovery of an optic pathway glioma (OPG), a pathology more classically expected in pediatric cohorts, highlights the value of maintaining continuous clinical and radiological vigilance throughout adulthood. Furthermore, the long-term monitoring of such lesions can be significantly enhanced by integrating multimodal diagnostic tools. Specifically, macular ganglion cell layer (GCL) analysis alongside peripapillary retinal nerve fiber layer (pRNFL) structural assessments represents a highly sensitive screening adjunct capable of detecting subclinical structural changes before global axonal loss or functional visual field deficits manifest.

The co-occurrence of a rare subcutaneous venous aneurysm further enriches the documented clinical spectrum of NF1-associated vascular abnormalities. Rather than a definitive systemic manifestation, this finding underscores the structural variability of the disease's vasculopathy, which remains a critical yet sometimes overlooked component. Additionally, the atypical persistence of T2-hyperintense foci, or unidentified bright objects (UBOs), at 49 years of age serves as a notable radiographical feature. Within the framework of current literature, recognizing these benign white matter abnormalities and their long-term evolution is essential for clinicians to avoid misdiagnosing them as ischemic or inflammatory pathologies in older patients. Ultimately, while the underlying phenotypic expressivity is proposed in academic literature to be driven by the constitutional hyperactivation of the RAS/MAPK pathway, this case clinically reinforces that optimizing the long-term management of both common and rare manifestations in adult NF1 patients necessitates a proactive, multidisciplinary approach.
